# Supplementary material for: Liganded T3 receptor β2 inhibits the positive feedback autoregulation of the gene for GATA2, a transcription factor critical for thyrotropin production
Source: PLoS One. 2020 Jan 15;15(1):e0227646. doi: 10.1371/journal.pone.0227646 (PMC6961892; doi:10.1371/journal.pone.0227646)
Supplement: S1 Raw Images — (PDF) [file pone.0227646.s001.pdf]

20100604 T3+/- GATA western blotting exp.1

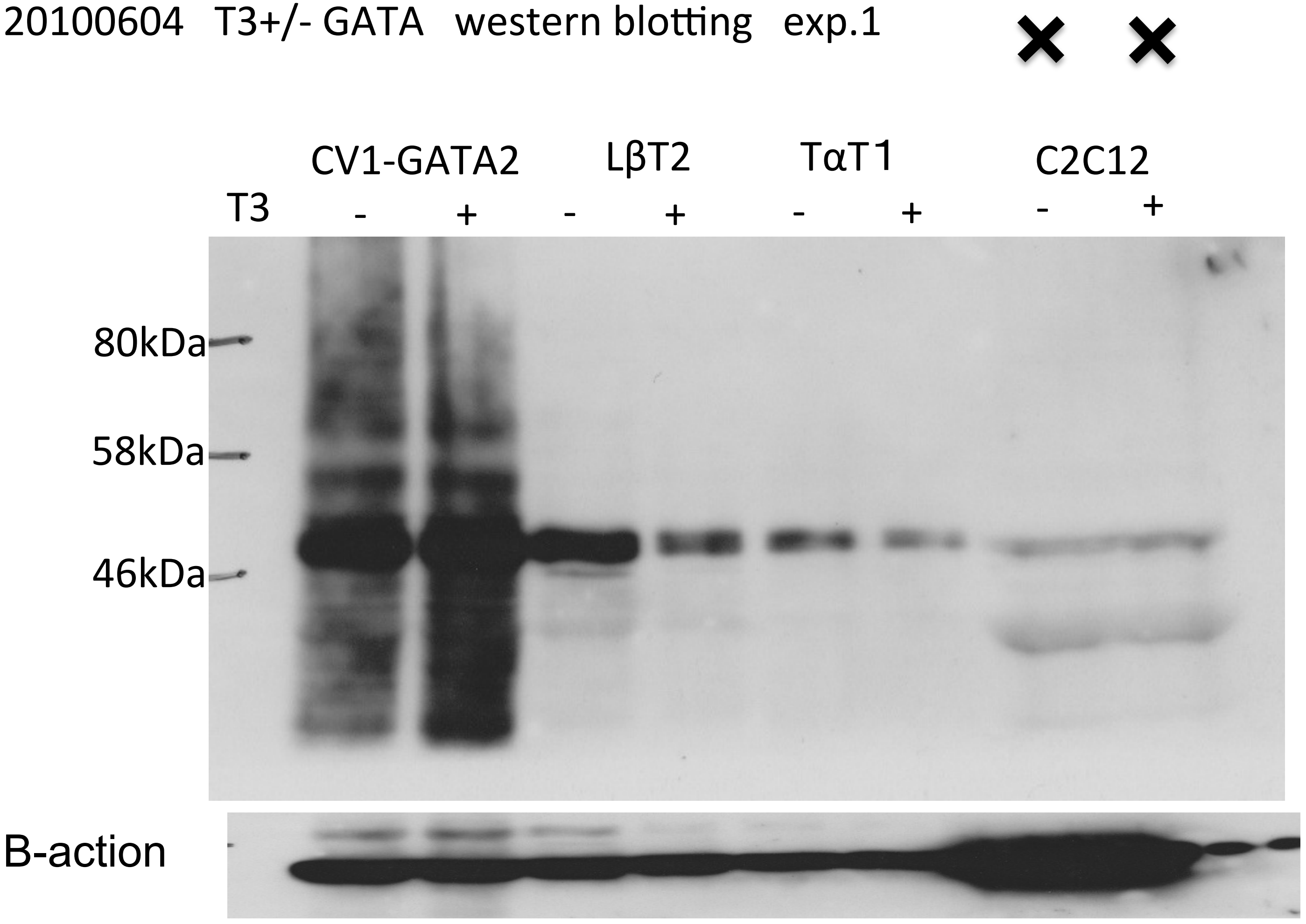

Fig. 4A

20100422 LβT2 TRb2 expression

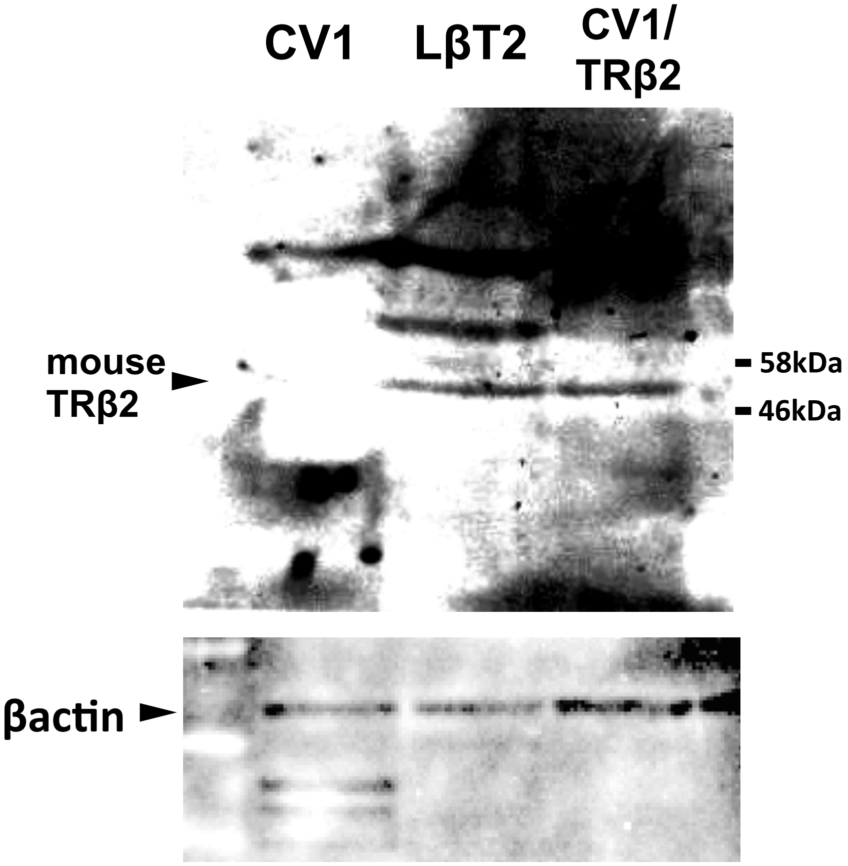

Fig. 4B

20100716 LβT2 FLAG-GATA expression western blotting

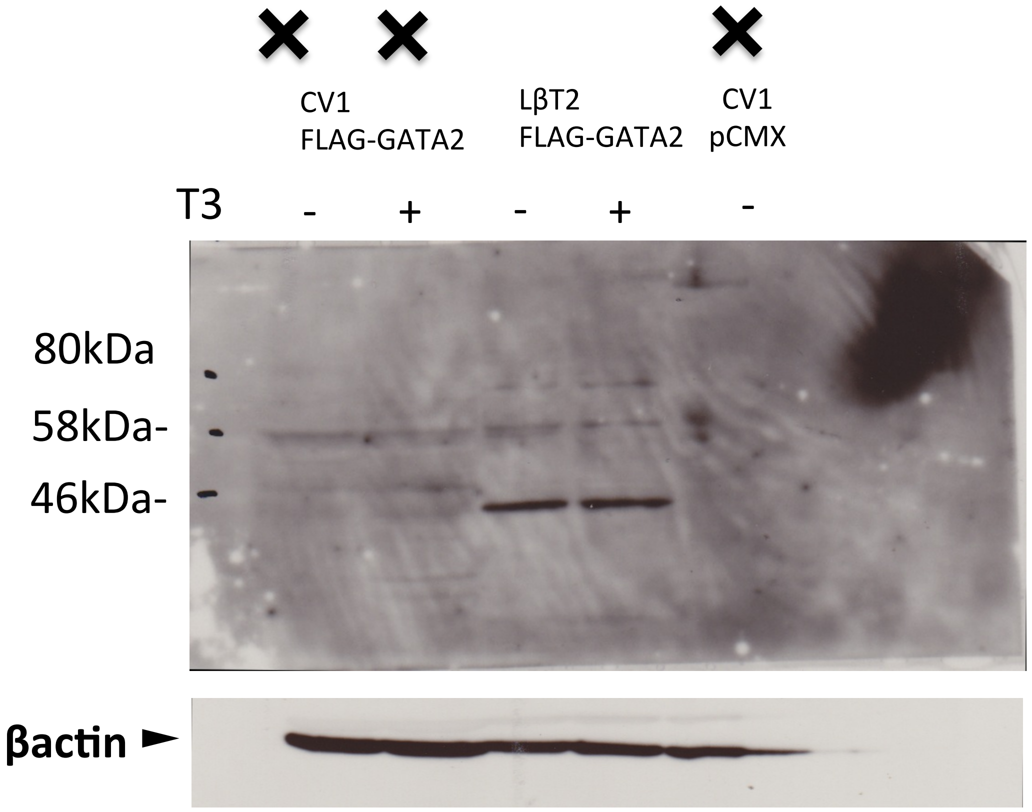

Fig. 4C

20101008 LβT2 TPAdose GATA expression western blotting

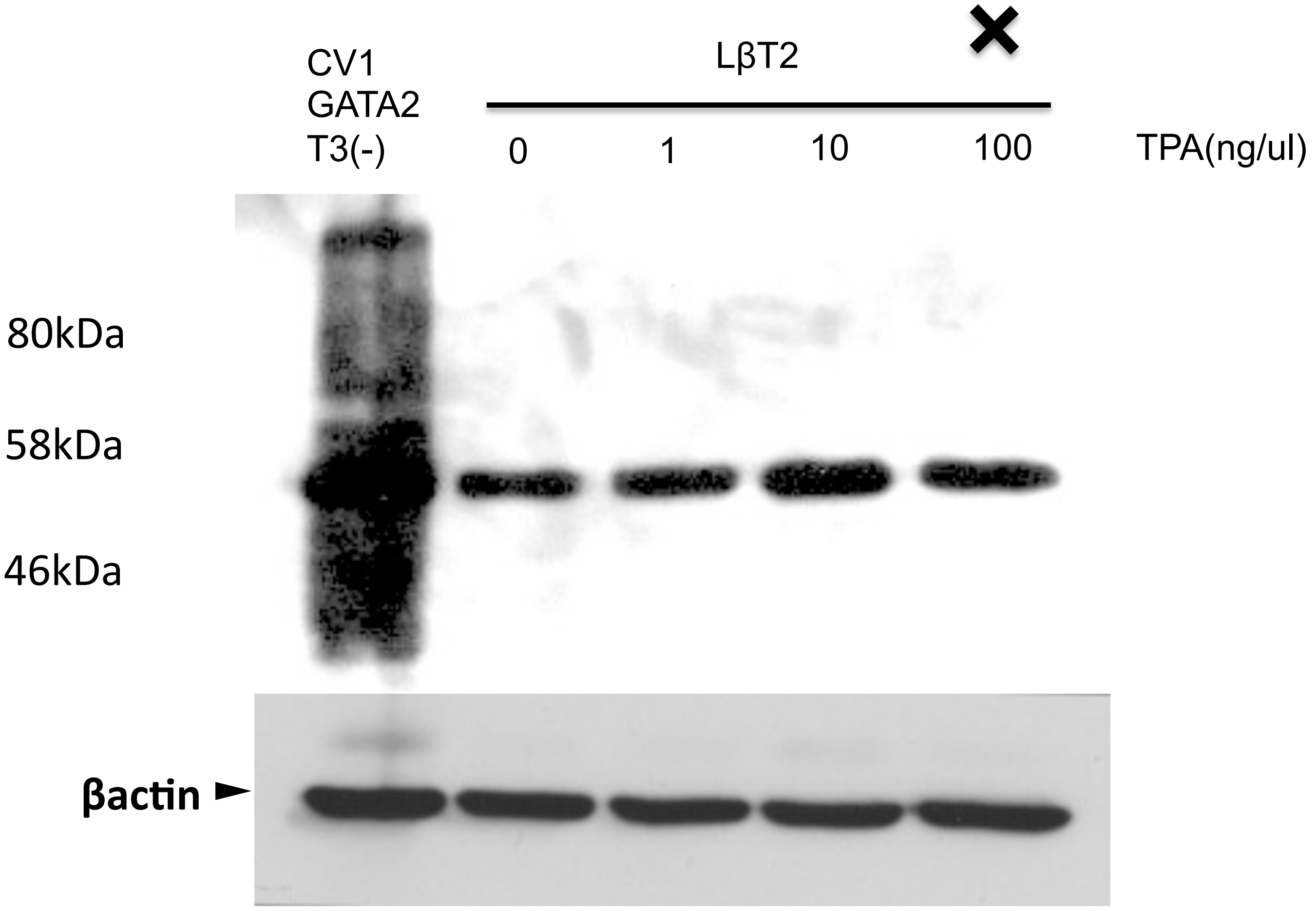

Fig. 4D

20100922 LβT2 T3dose GATA expression western blotting

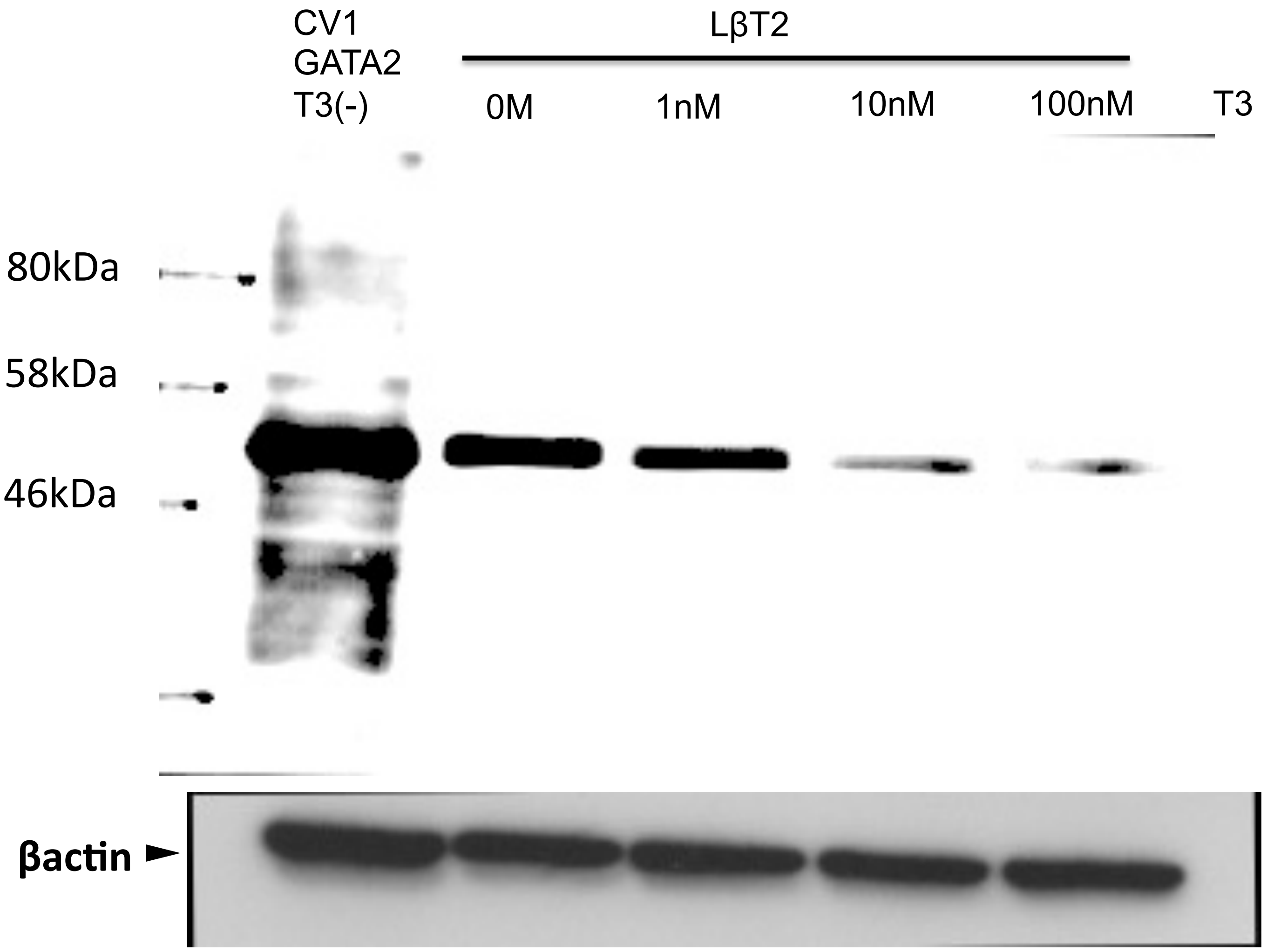

Fig. 5A

2010112 LβT2 T3(+) time response GATA expression western blotting

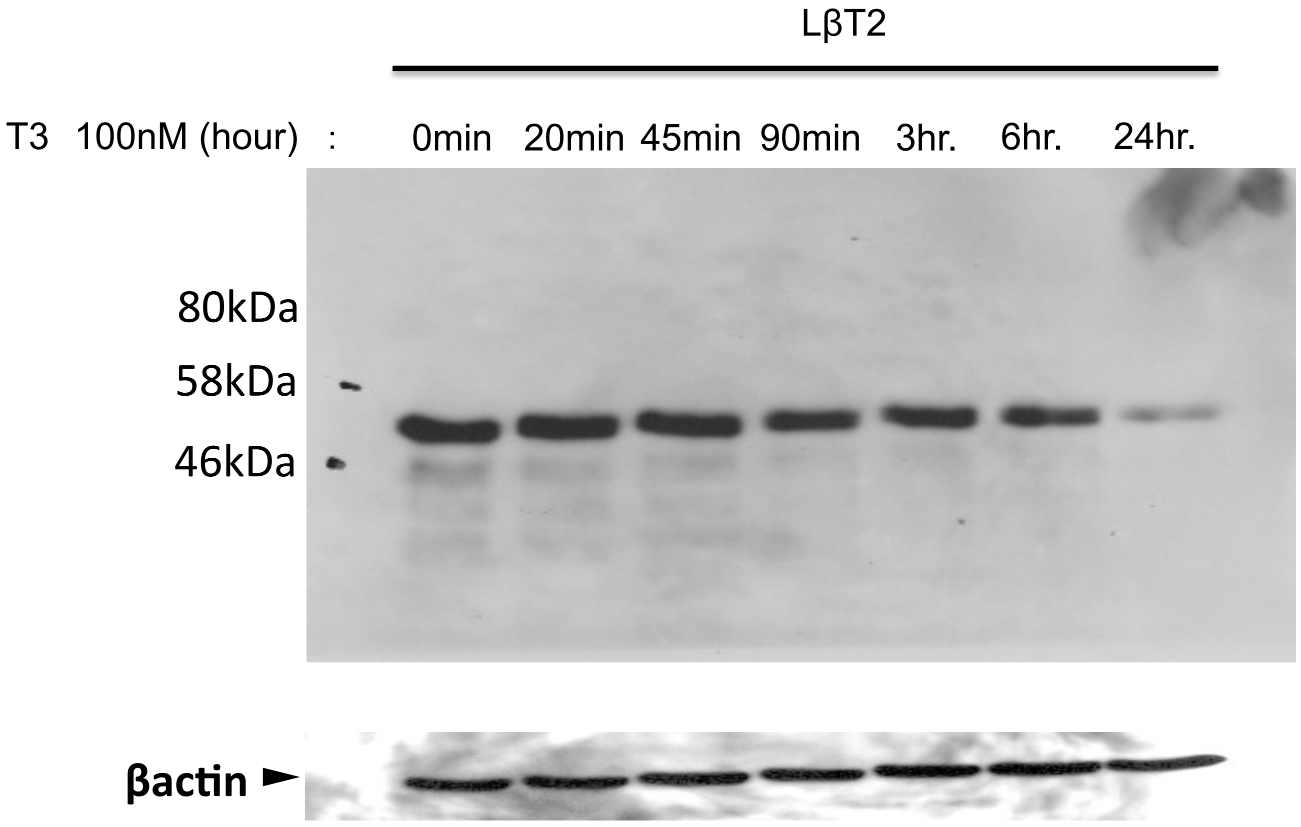

Fig. 5B

20110323
MG132 1μM   Anti- GATA time response westernblotting

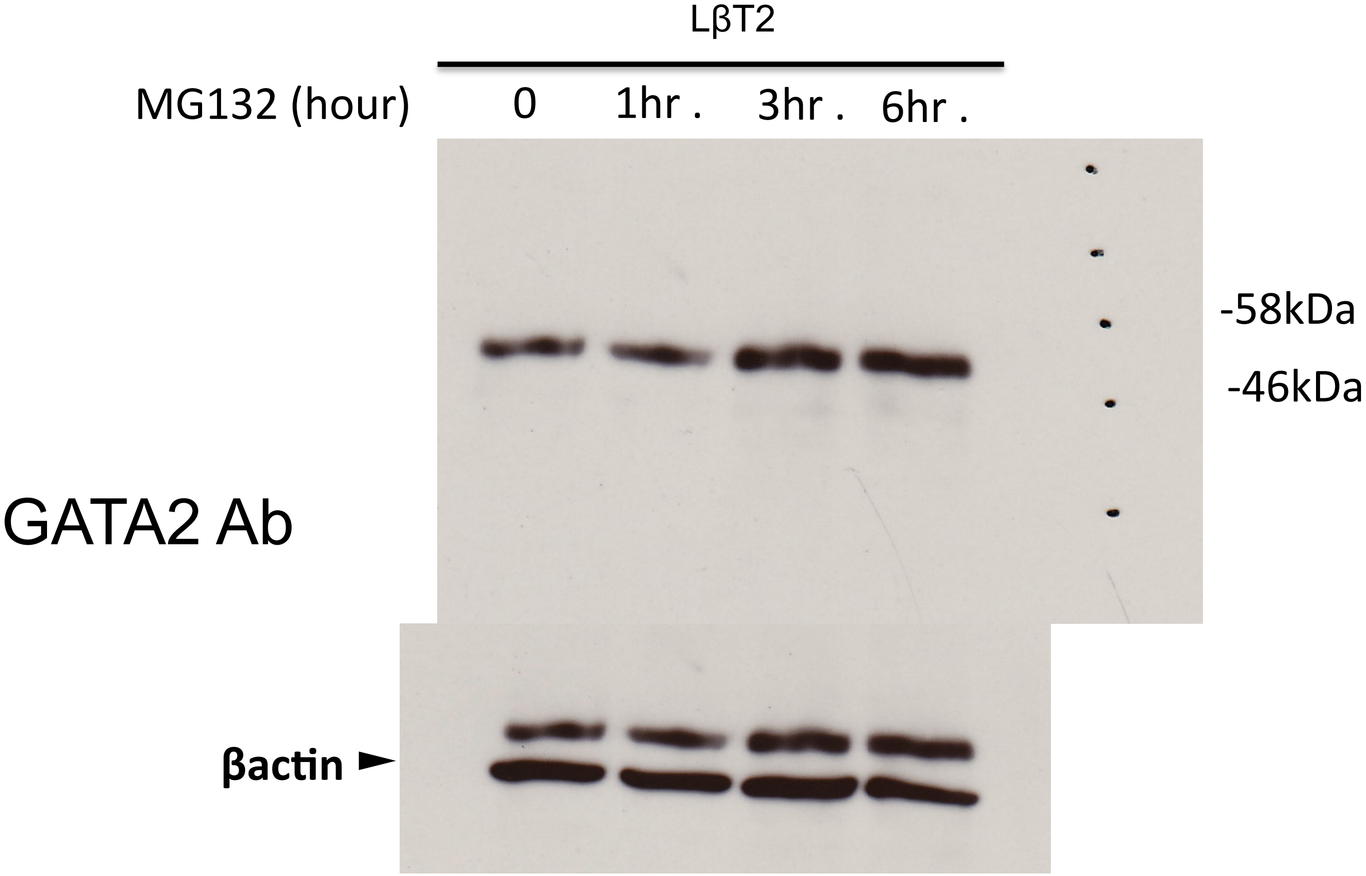

Fig. 5C

20110223

LβT2 T3+MG132 Anti- GATA westernblotting

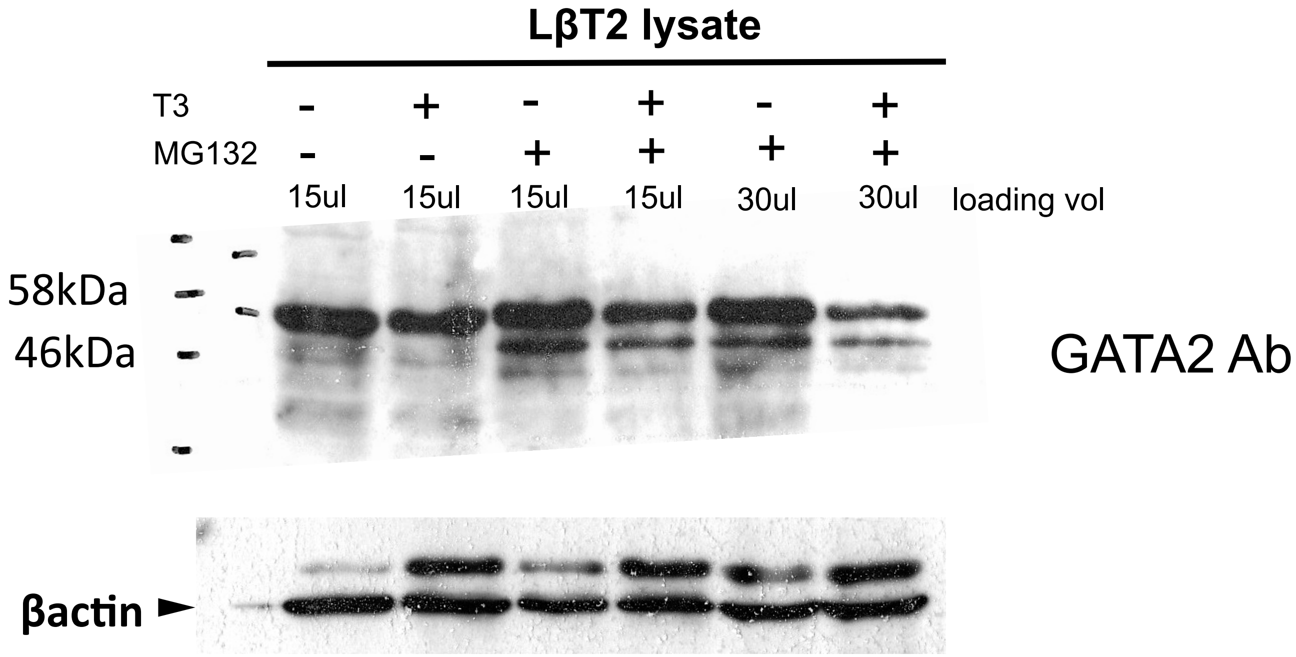

Fig. 5D
