# Supplementary material for: Liganded T3 receptor β2 inhibits the positive feedback autoregulation of the gene for GATA2, a transcription factor critical for thyrotropin production
Source: PLoS One. 2020 Jan 15;15(1):e0227646. doi: 10.1371/journal.pone.0227646 (PMC6961892; doi:10.1371/journal.pone.0227646)
Supplement: Attachment — Submitted filename: PLoSOneRebuttal.docx [file pone.0227646.s002.docx]

REFERENCE: PONE-D-19-22945
Title: "Liganded T3 receptor β2 inhibits the positive feedback autoregulation of the gene for GATA2, a transcription factor critical for thyrotropin production”

AUTHORS: Naoko Hirahara and Hiroko Misawa Nakamura et al.

PLOS ONE

Academic Editor: Hiroyoshi Ariga

Please re-evaluate our revised manuscript. Although our previous manuscript was screened once by a native English speaker before first submission, the referee recommended us to edit it again regarding in particular to the usage of articles. So we asked another (more experienced) person to re-check it. Based on his recommendation, we deleted “the” from “the thyrotropin production” in the title. If it can be now acceptable in your journal, we would be very much pleased. Thank you for your kind arrangement.

Shigekazu Sasaki. MD.

Senior Assistant Professor

Second Division of Internal Medicine

Hamamatsu University School of Medicine

1-20-1 Handayama Higashi-ku

Hamamatsu 431-3192, Japan

E-mail: sasakis@hama-med.ac.jp

RESPONSE TO THE COMMENTS FROM REVIEWER

Major.
Regarding to the effect of T3 on the CAT reporter genes co-transfected with vector only and GATA2 only (Fig.2),

To evaluate the effect of T3 (100 nM) on the activities of CAT reporter genes co-transfected with vector only and GATA2 only, we conducted additional CAT assays for five times independently. Using the value of the activity of the CMV-based CAT reporter gene (inter assay control) as 100, we combined these data with the original data of Fig. 2A and created new Fig. 2A. We did not find any statistical significance in CAT activities between absence or presence of T3 when we co-transfected vector only or GATA2 only.

Minor.
Regarding to “has not performed” of p. 3 L83 in the previous manuscript,

we added “been”.

Regarding to period for “hr” in p.6 L190, p.7 L198, p.7 L201 and p.7 L222 of the previous manuscript,

we deleted all the periods for “hr”.

Regarding to lack of a closing bracket in L225-227 of the previous manuscript,

we added a closing bracket.

Regarding to “theoretically” in p.9 284 of the previous manuscript,

we rephrased the sentence as follows.
Using the experimental system in Fig. 2B, we wanted to know whether the liganded TR functions as a transcriptional repressor that was defined by the theory of multi-dimensional in put function proposed by Uri Alon (Fig. 3 in set) [49]. We co-transfected various amounts of---.

Regarding to “ as log scale” in the previous manuscript,

we corrected “ as log scale” to “ in a log scale”

Regarding to lacking or inappropriate usage of articles,

Thank you for your recommendation. Although a native English speaker screened our previous manuscript once before submission, we asked another (more experienced) person to re-check English including usage of articles. He pointed a lot of mistakes in the previous manuscript. We corrected all of them. In addition, based on his recommendation, we deleted “the” from “the thyrotropin production” in the title (Please see “Track Changes”). We appreciate your recommendation very much.

Regarding to “Several mechanisms” in p.14, l430 of the previous manuscript,

We altered the sentences as follows.

Third, T3 destabilizes the TSHβ mRNA but not CGA mRNA [78]. Fourth, T3 may inhibit the expression of Pit1 [79], which is a critical transcription factor for the TSHβ gene (Fig.7), but not the CGA gene [2]. Finally, the cell cycle of thyrotrophs [80, 81] may also be involved because sustained hypothyroidism often causes thyrotroph hyperplasia, resulting in the massive enlargement of the anterior pituitary [82].

In the course of this revision, we found two mistakes in p.18 L554 and L556: the phrase “the CAT activity” in these sentences was corrected to “the GATA2/GAPDH ratio”.
