# Supplementary material for: Liganded T3 receptor β2 inhibits the positive feedback autoregulation of the gene for GATA2, a transcription factor critical for thyrotropin production
Source: PLoS One. 2020 Jan 15;15(1):e0227646. doi: 10.1371/journal.pone.0227646 (PMC6961892; doi:10.1371/journal.pone.0227646)
Supplement: Attachment — Submitted filename: PLoSOneRebuttalR011218.docx [file pone.0227646.s003.docx]

REFERENCE: PONE-D-19-22945

Title: "Liganded T3 receptor _2 inhibits the positive feedback autoregulation of the gene for GATA2, a transcription factor critical for thyrotropin production”

AUTHORS: Naoko Hirahara and Hiroko Misawa Nakamura et al.

PLOS ONE

Academic Editor: Hiroyoshi Ariga

Please re-evaluate our revised manuscript. We corrected the sentences that the referee pointed. In addition, we transferred the original figure file to seven separate tif-style files using PACE home page (https://pacev2.apexcovantage.com/). If it can be now acceptable in your journal, we would be very much pleased. Thank you for your kind arrangement.

Shigekazu Sasaki. MD.

Senior Assistant Professor

Second Division of Internal Medicine

Hamamatsu University School of Medicine

1-20-1 Handayama Higashi-ku

Hamamatsu 431-3192, Japan

E-mail: sasakis@hama-med.ac.jp

RESPONSE TO THE COMMENTS FROM REVIEWER

We corrected the sentences that the referee pointed as follows.

Line 43, “a determinants”

We corrected as “ a determinant”

Line 288, “ wanted to know”

We corrected as “aimed to clarify”.
